# Defining the Scale to Build Complex Networks with a 40-Year Norwegian Intraplate Seismicity Dataset

**DOI:** 10.3390/e25091284

**Published:** 2023-08-31

**Authors:** Claudia Pavez-Orrego, Denisse Pastén

**Affiliations:** 1 SINTEF Industry, S.P. Andersens Vei 15B, 7031 Trondheim, Norway; 2 Departamento de Física, Facultad de Ciencias, Universidad de Chile, Las Palmeras 3425, Ñuñoa, Santiago 7800003, Chile; denisse.pasten.g@gmail.com

**Keywords:** complex networks, intraplate seismicity, earthquake distribution

## Abstract

We present a new complex network-based study focused on intraplate earthquakes recorded in southern Norway during the period 1980–2020. One of the most recognized limitations of spatial complex network procedures and analyses concerns the definition of adequate cell size, which is the focus of this approach. In the present study, we analyze the influence of observational errors of hypocentral and epicentral locations of seismic events in the construction of a complex network, looking for the best cell size to build it and to develop a basis for interpreting the results in terms of the structure of the complex network in this seismic region. We focus the analysis on the degree distribution of the complex networks. We observed a strong result of the cell size for the slope of the degree distribution of the nodes, called the critical exponent γ. Based on the Abe–Suzuki method, the slope (γ) showed a negligible variation between the construction of 3- and 2-dimensional complex networks. The results were also very similar for a complex network built with subsets of seismic events. These results suggest a weak influence of observational errors measured for the coordinates latitude, longitude, and depth in the outcomes obtained with this particular methodology and for this high-quality dataset. These results imply stable behavior of the complex network, which shows a structure of hubs for small values of the cell size and a more homogeneous degree distribution when the cell size increases. In all the analyses, the γ parameter showed smaller values of the error bars for greater values of the cell size. To keep the structure of hubs and small error bars, a better range of the side sizes was determined to be between 8 to 16 km. From now on, these values can be used as the most stable cell sizes to perform any kind of study concerning complex network studies in southern Norway.

## 1. Introduction

The analysis of the distribution and physical behavior of earthquakes in Norway has been approached from different points of view, which include, for example, imaging studies [1,2,3,4,5,6,7,8,9,10,11,12,13], the physics of earthquakes [14], seismic hazards [15,16], and tectonics and seismology [14,17,18,19]. Recently, a whole new area regarding complex networks and fractality has been investigated, which aims to build a deeper understanding of the connections between seismic events in time and space [20].

Complex networks are able to show non-trivial behavior of physical systems through the analysis of their topological features. They can be categorized into many different types, such as random complex networks [21], small-world behavior [22], which indicates the need to take just a small number of steps to go from one node to another one, and scale-free behavior [23], which shows the structural organization of a system. Complex networks have been developed and applied in the study of different systems that show non-trivial topological behavior, such as biology [24,25,26], economics [27,28] or social relationships [29,30]. The application of complex networks in geophysics has grown in recent years, providing a new perspective in the Earth sciences [31,32,33,34,35,36,37,38,39,40,41].

In particular, over the last twenty years, complex networks have been used to analyze the complex behavior of earthquake distribution in time and space. In this sense, many studies have been carried out. For example, building networks based on the spatial position of earthquakes, their epicenters or hypocenters [39,42,43,44], or following their time series [45,46], has included application of the visibility graph method (VG) to earthquakes in Italy, finding that the probability distribution of connectivity follows a power law. Moreover, Telesca et al. (2020) [47], applied a horizontal visibility graph (HVG) to determine changes in the reversibility of the time series measured for the Iquique earthquake (Chile, 2014), showing that they were reversible for the dataset without the aftershocks and irreversible for the catalog with aftershocks.Therefore, earthquakes have been studied through complex networks mainly in two different ways: (1) based on following temporal sequences or (2) based on the spatial distribution of seismic events. One of the most recognized approaches is that developed by Abe and Suzuki (2006) [39], whose method involves the building of spatial complex networks with earthquake datasets. The method is based on the size of a cubic or a plane cell: if the cell contains a hypocenter (cubic) or an epicenter (plane), a network node is defined. Then, the network can be based on the dimension of the cubes used to divide the three-dimensional space (latitude, longitude, and depth). In this sense, any attempt to define complex networks on these kinds of systems will require a suitable and stable cell size as the network topology and its physical properties depend on it.

In this study, we build a complex network with earthquakes recorded in southern Norway, using the above-described method of Abe and Suzuki, (2006) [39]. Our purpose is to define a cell size range independent of the measurement errors generally associated with hypocenter and epicenter estimations, with the aim of gaining a better understanding of scale effects in the complex network. This will be carried out by testing the stability of the values obtained for the critical exponent γ in directed complex networks built with an earthquake dataset measured in southern Norway and with temporal and spatial subsets. We also analyze the influence of the measurement errors in the size of the chosen cell in the degree distribution. As a main result, we obtain a range of cubic cell sizes that show independence of the measurement errors and for which the construction of a complex network will be reliable.

## 2. Intraplate Seismicity in Southern Norway

Seismicity rates in Norway are the highest in northern Europe [17]. Seismic events occur periodically (NNSN report, 2018) [48], with low to intermediate magnitudes ($M_{L}\leq 4.0$). Natural earthquakes are strictly intraplate, both onshore and offshore, at the passive continental margin [17,49].

**Figure 1:**
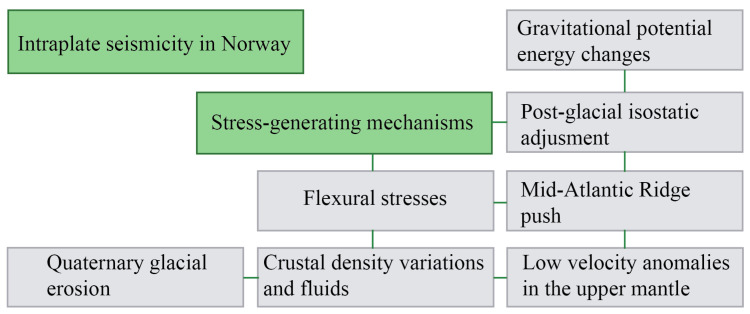
Summary of the main stress generating mechanisms for intraplate seismicity in Norway.

It is commonly accepted by the seismological community that this intraplate activity emerges due to a combination of stress-generating mechanisms, which include crustal to local scale (Olesen et al., 2013): (i) gravitational potential energy changes produced due to topographic loads, (ii) post-glacial isostatic adjustments, (iii) Mid-Atlantic ridge push (iv) Quaternary glacial erosion (v) flexural stresses through sedimentation [17], (vi) crustal density variations, and spatial coincidence with anomalous low-velocity zones of seismic waves in the upper mantle [50,51,52] (Figure 1).

**Figure 2:**
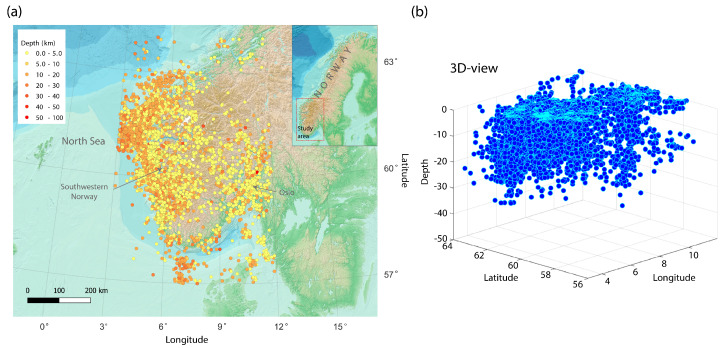
(**a**) Seismicity map with local events. Color legend shows earthquakes arranged by depth. The study area is marked with a red box in the regional inset. (**b**) 3D−view of hypocenters, showing in−depth earthquake distribution.

Concerning the spatial earthquake location, the highest seismicity levels occur in the rifted continental margin as well as in the strongly faulted regions near to the rifts in the North Sea, and in the coast of south-western Norway [18,48]. This area, in which we focus this study, also presents high amounts of seismic events on the mainland in a zone that is highly influenced by the post-Caledonian faults [53]. Most of the seismic events are located in the upper 20 km of the crust [17,18] (Figure 2). Meanwhile, deep earthquakes occur mainly offshore, dominated by reverse faulting and the Mid-Atlantic Ridge push. On the other side, shallow earthquakes occur onshore, where normal faulting is dominant and the horizontal tension is coast perpendicular [54].

## 3. Data

The seismic catalog for the period 1980–2019 was downloaded from the EPOS Norway portal, the European Plate Observing System https://epos-no.uib.no/eposn-data-portal/ (accessed on 12 January 2023). To the present day, the database offers a list of 40 possible data services. This list includes access to the seismological data offered by the Norwegian National Seismic Network. The direct way to access the data is shown in Appendix A. The EPOS-N project is research-based, and is focused on understanding of the Earth’s deformational processes, geohazards, and georesources. The project is collaborative, with many Norwegian institutions continuously contributing geological and geophysical datasets to the data portal: the University of Bergen (coordinator), the University of Oslo, the Geological Survey of Norway, NORSAR, the Norwegian Mapping Authority, and the Christian Michelsen Research Centre.

The original catalog consisted of 77,622 global seismic events. This catalog was downloaded as an Excel sheet (see Appendix A) and contains origin time and hypocentral location with their respective errors, magnitude and magnitude type, number of stations, rms, azimuthal gap, strike, dip, and rake. More details about the construction process of this catalog can be found in the 2018 and 2019 NNSN annual reports (NNSN annual report, 2018–2019) [48,55].

**Figure 3:**
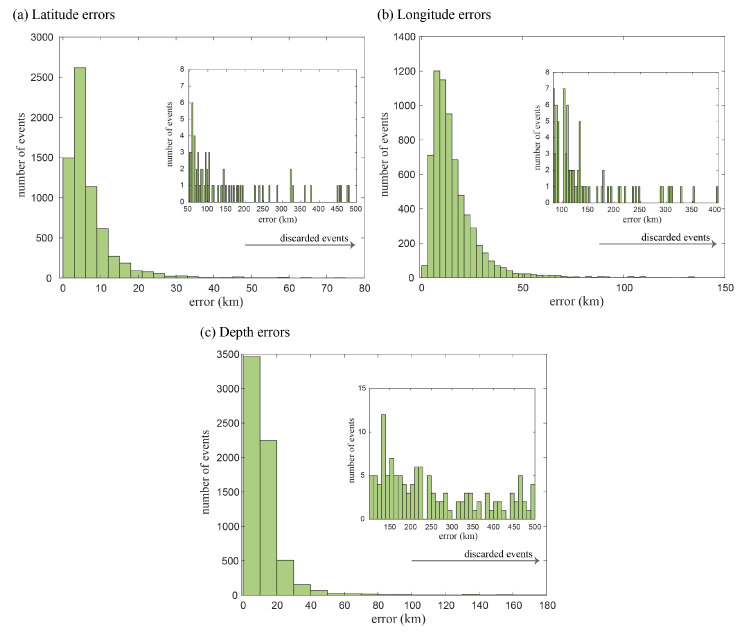
(**a**–**c**) Histograms with location errors in latitude, longitude and depth, respectively.

**Figure 4:**
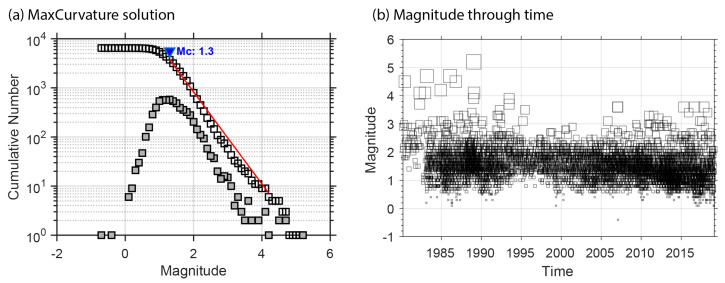
(**a**) Magnitude of completeness (blue triangle), calculated through the maximum curvature method. Gray and white squares represent the cumulative and discrete number of events, respectively. (**b**) Magnitude distribution vs. time.

With the aim of selecting the relevant information for our study, the catalog was initially filtered for southern Norway, considering the area between 3${}^{\circ }$ and 12${}^{\circ }$ E and 57${}^{\circ }$ and 64${}^{\circ }$ N. Large hypocentral errors were observed in the catalog, which might be explained as a consequence of picking errors or large azimuthal gaps for events with a high number of observations. To avoid this, we consider a minimum number of recording stations equal to six. With this number, we also prevent incorrect low errors based on a low number of observations, where the location solutions might fit with small residuals. Based on the distribution of latitude, longitude, and depth errors, the last filtering consisted of the inspection of the histogram, from where out-of-average events—48, 81, and 100 km error locations for the y, x, z axes—were excluded from the curve (Figure 3). The spatial, error, and distribution filters resulted in a total of 6469 earthquakes for further analysis (Figure 2 and Figure 3). Moreover, the catalog was filtered by its magnitude of completeness $M_{c}=1.3$. After this process, the total number of events was 3739 (Figure 4a,b).

## 4. Methodology

We built a complex network using the Abe and Suzuki [39] method applied over a seismic dataset measured in southern Norway during the period 1980–2020, i.e., we used their hypocenter coordinates and the time sequence as inputs. To perform the spatial analyses, we then used the spatial coordinates (longitude, latitude, depth) in kilometers. The latitude is represented by the angle θ, and the longitude is represented by the angle ϕ. This conversion is performed by using the following expressions:

(1) $$d_{i}^{NS}=R\left(\theta_{i}-\theta_{0}\right),$$

(2) $$d_{i}^{EW}=R\left(\phi_{i}-\phi_{0}\right)\cos\left(\theta_{av}\right),$$

(3) $$d_{i}^{z}=z_{i},$$

where $z_{i}$ is the depth and $\theta_{av}$ is the average latitude, $\theta_{0}$ and $\phi_{0}$ are the minimum values for the latitude and longitude, and *R* is the radius of the Earth, assumed for this study to be 6370 km.

**Figure 5:**
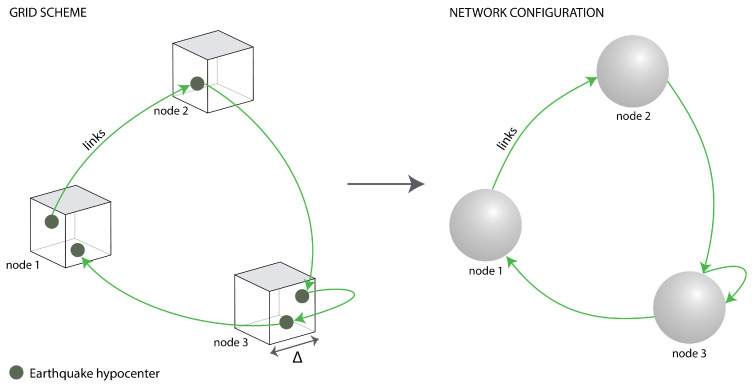
Schematic representation of the cubic cells representing nodes in the complex network. The cube side size is Δ.

A complex network consists of **nodes** connected between them through **links**. Once we have converted the spatial coordinates (longitude, latitude, depth) into kilometers, we can build the complex network with the seismic dataset. To follow the method of Abe and Suzuki (2006) [39], we must define what a node is. For this, we divide each zone into cubic cells with a side size between Δ=5 km and Δ=20 km. Then, we check if one or more hypocenters are inside the cubic cell; if so, the cell is called a node. Then, we place the connections between the nodes following the temporal sequence of the seismic events. The direction of the connections between the nodes is defined through the temporal sequence of the seismic events in the region, as shown in Figure 5. Consequently, a directed network following the Abe–Suzuki method [39] is built.

Then, we place the connections between the nodes following the temporal sequence of the seismic events, so the order of events is preserved as in the standard time analysis [56,57,58]. The direction of the connections between nodes is defined through the temporal sequence of the seismic events in the region, as shown in Figure 5.

A complex network can be characterized by different metrics. Among them, it is important here to mention the clustering coefficient (*C*), which measures the tendency of the nodes to form triangles (clusters) between them, the path length (*L*), defined as the average of the shortest path length in the network, and the degree $k_{i}$, which represents the number of connections of the node *i*. Good examples regarding metrics applications can be found, for example, in Watts and Strogatz (1998) [22], who defined the term “small world” for complex networks, following certain metric criteria imposed over *L* and *C*. Also, Barabási and Albert, (1999) [23] defined “scale-free” behavior of a complex network when the probability of the degree $k_{i}$ is a power law in a log-log plot.

Now, once we have built the directed complex network for a one cubic cell side value, we focus on a complex network basic measure: the degree of the node. We analyze the behavior of the critical exponent γ, which corresponds to the slope of the degree distribution. For earthquake datasets, this probability is represented by a power law [39,40,41,42,43],

(4) $$P\left(k\right)∼k^{-\gamma },$$

where *k* is the degree of the nodes and γ is the slope of the distribution in a log-log plot.

To understand the response of the γ exponent in different scenarios, the analyses are carried out over both the prefiltered and the complete datasets (without and with $M_{c}$). For the first case, we analyze the entire catalog (6469 events), and the first half of it (3235 events). For the second case, we separate the dataset into two regions: the western zone (longitude 3.0° to 7.5° E, with 3111 seismic events) and the Oslo region (longitude 7.5° to 12.0° E, with 628 seismic events).

## 5. Results

### 5.1. Prefiltered Dataset

**Figure 6:**
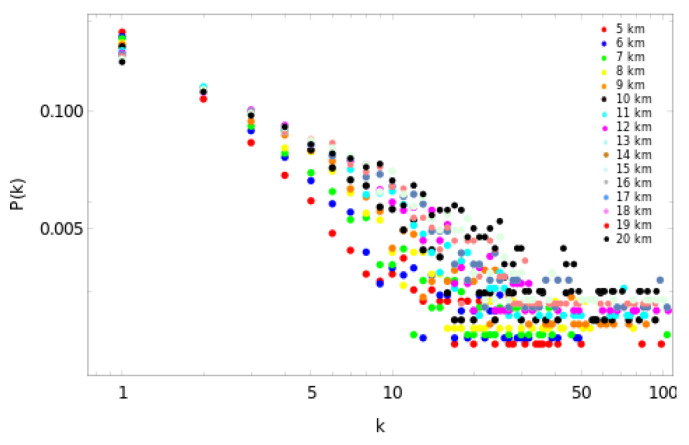
Log-log plot of the degree distribution for the studied complex networks. The complex networks were built with values of the side between Δ= 5 km and Δ= 20 km. The scale-free behavior is clear.

**Figure 7:**
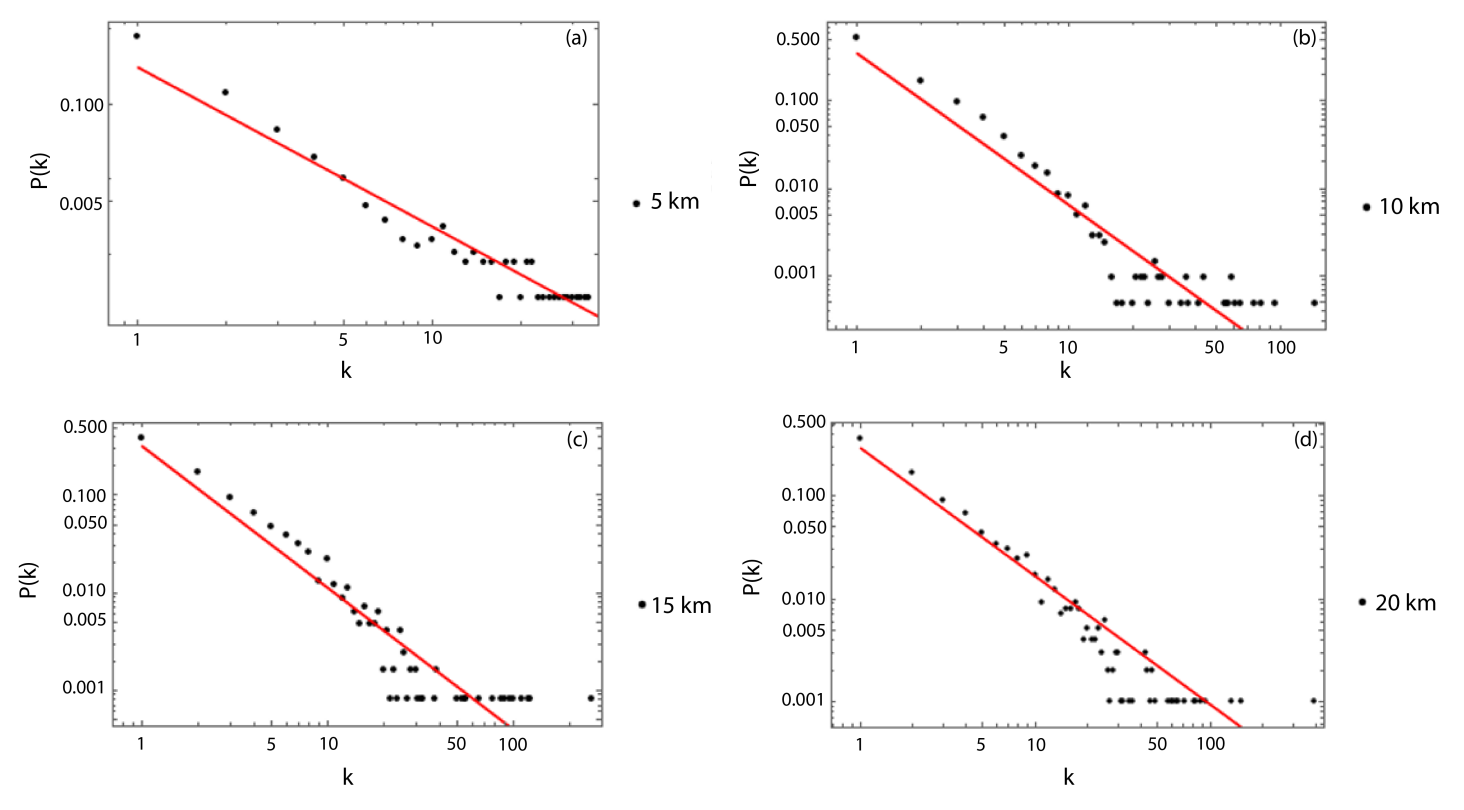
Examples of the scale-free behavior of the degree distribution of nodes with their respective linear fits. (**a**) Degree distribution for Δ= 5 km with γ= 2.1 ± 0.1. (**b**) Degree distribution for Δ= 10 km with γ= 1.7 ± 0.1. (**c**) Degree distributions for Δ= 15 km with γ= 1.45 ± 0.08. (**d**) Degree distribution for Δ= 20 km with γ= 1.24 ± 0.05.

As a first result, we show the scale-free behavior for the degree distribution of the dataset without the magnitude of the completeness filter, and, for all the used sizes of Δ, which vary from 5 to 20 km (Figure 6). We choose those values of the cell side size to have a sufficient number of cubic cells to analyze. As particular examples, Figure 7a–d show the scale-free behavior and the adjustment of the slopes for Δ=5,10,15 and 20 km, respectively. In order to calculate the best fit for each degree distribution, we considered the same $k_{min}=1$ for all the cubic cell sizes, and we neglected between one to three points of the tails. We then computed their respective slopes, which represent the critical exponent γ. From Figure 7, it is possible to observe a better fit of the slope from Δ=10 km, being the best fit for Δ= 20 km. The values of γ, together with their respective errors, are listed in Table 1.

**Figure 8:**
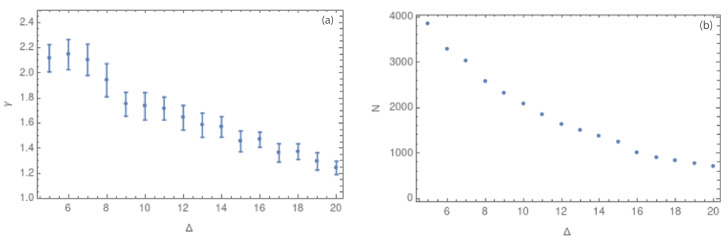
(**a**) Values of the critical exponent γ for side sizes Δ between 5 and 20 km. (**b**) Values of the number of nodes *N* for the same range of Δ.

After corroborating that all the complex networks show scale-free behavior, we focus on the γ value. Figure 8a,b show the values of γ and the number of nodes, respectively, for different values of the cubic cell side, considering a range of Δ from 5 to 20 km. Figure 7a–d and Figure 8a show that smaller values of Δ have few nodes with a large degree, implying that the slope of the degree distribution is large (close to 2.0). On the other hand, values of Δ close to 20 km show a more homogeneous degree distribution, with a lower value of γ (close to 1.2). We can interpret these results in dependence of the cubic cell size as follows: with side sizes close to 20 km, the number of contained hypocenters is higher, so the degree distribution is more homogeneous. The error bars from the linear fit of the degree distribution decrease as the cubic cell side grows. We can also notice from Table 1 that the error bars decrease from Δ= 14 km.

**Figure 9:**
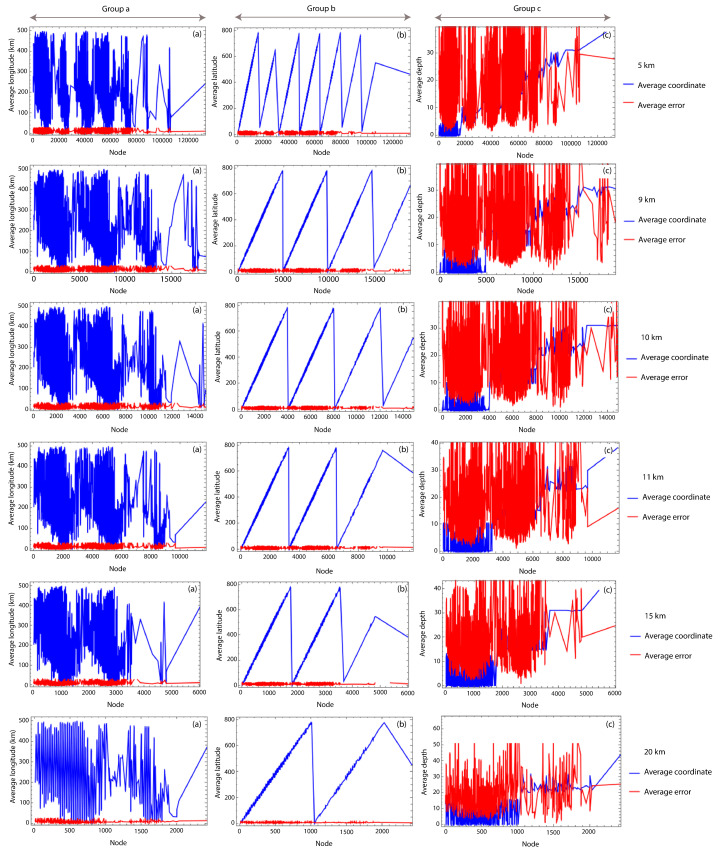
Values of the average coordinates in longitude (group (**a**)), latitude (group (**b**)), and depth (group (**c**)) and associated errors. These plots correspond to Δ equal to 5, 9, 10, 11, 15, and 20 km, from top to bottom. All averages are in km.

The earthquake dataset measured in southern Norway is of high quality, with associated errors for each hypocentral location. This allows us to include the average hypocentral errors in kilometers for all nodes in longitude, latitude, and depth. As an example, Figure 9 shows the average errors for Δ= 5, 9, 10, 11, 15, and 20 km, respectively. Specifically, groups (a), (b) and (c) in Figure 9 show a comparison between the average hypocentral longitude, latitude, and depth in km, with their respective average errors.

**Figure 10:**
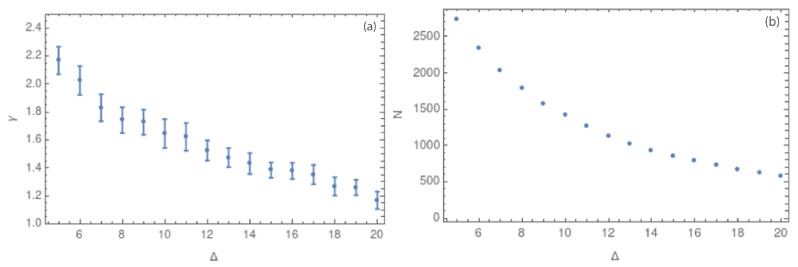
(**a**) Values of the critical exponent γ in 2 dimensions for Δ between 5 and 20 km. (**b**) Values of the number of nodes *N* for each value of Δ between 5 and 20 km.

Figure 3 and Figure 9 show the measurement errors in latitude, longitude, and depth. From Figure 3, we can observe that the largest errors in both latitude and longitude are concentrated in a few measurements, while most of the data have low errors (less than 15 km). Meanwhile, Figure 9 shows that the average error by node in the coordinates’ longitude and latitude is negligible. Nevertheless, the errors in depth are considerable. The percentage of seismic events with errors greater than 20 km is less than the 4%, 23%, and 12% in latitude, longitude, and depth, respectively. As a consequence of this, we analyze the stability of the values of γ for a 2-dimensional complex network, i.e., using only the coordinates of longitude and latitude. This result is shown in Figure 10a,b.

**Figure 11:**
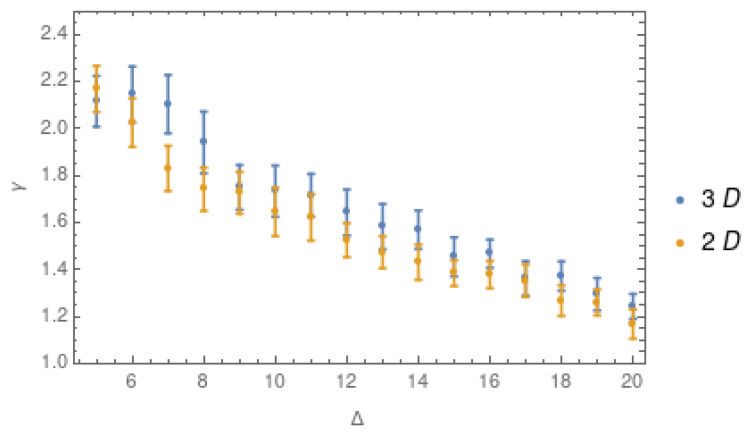
Values of the critical exponent γ in 3 dimensions (blue dots) and for 2 dimensions (yellow dots) for Δ between 5 km and 20 km.

Finally, we compare the results obtained in three dimensions (latitude, longitude, depth) and in two dimensions (latitude, longitude) in Figure 11. From this comparison, we observe a considerable similarity in the results for the value of γ in 2D and 3D, showing that the distribution of degrees is not affected by the change in dimension. This point is interesting because it means that the complex network keeps its behavior with few nodes with a large degree for small values of Δ, while the degree distribution evolves to a homogeneous distribution of the degree for larger values of Δ. In addition, we observe a decrease in the error bars for Δ values larger than 14 km, which suggests that this range is the best for applying any kind of analyses in complex networks for this tectonic environment.

#### Prefiltered Subset

A relevant step during complex network analyses is to check the results’ stability. To verify our first round of outcomes, we perform the same analysis by taking a subset of the entire catalog.

**Figure 12:**
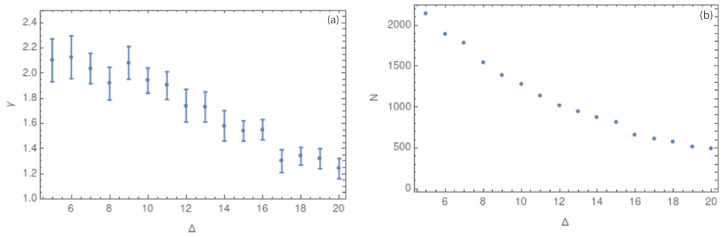
(**a**) Values of the critical exponent γ in 3 dimensions for Δ between 5 and 20 km (**b**) Values of the number of nodes *N* for each value of Δ between 5 and 20 km.

Figure 12a show the same behavior that we found for the first analysis: for small values of Δ, the complex networks have few nodes with a larger degree (high values of γ), whilst larger values of Δ give small values of γ (homogeneous degree distribution). We note how the error bars grow by using less seismic data.

### 5.2. Complete Dataset

**Figure 13:**
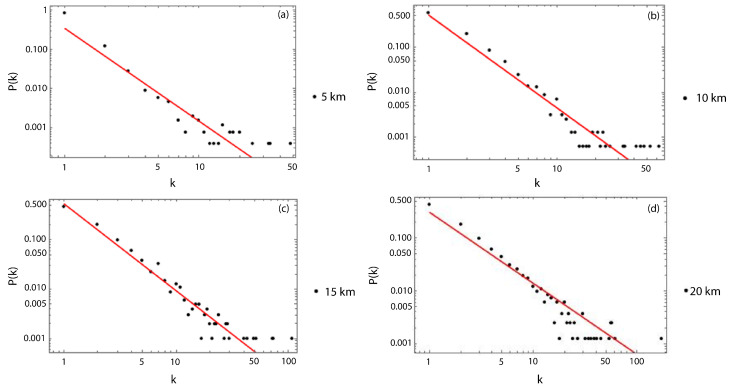
Examples of the scale-free behavior of the degree distribution of nodes with the respective linear fit for the dataset with the completeness magnitude. (**a**) Degree distribution for Δ= 5 km. (**b**) Degree distribution for Δ= 10 km. (**c**) Degree distribution for Δ= 15 km. (**d**) Degree distribution for Δ= 20 km.

The analyses must then be complemented using the seismic dataset with the magnitude of completeness $M_{c}=1.3$, which was computed in Section 3 (Figure 4). Figure 13 shows the scale-free behavior for the complex networks built with $M_{c}$.

**Figure 14:**
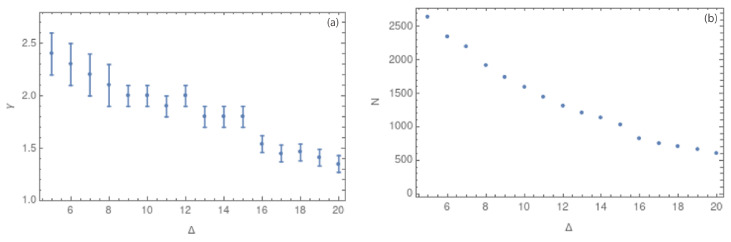
(**a**) Values of the critical exponent γ in 3 dimensions for Δ between 5 and 20 km (**b**) Values of the number of nodes *N* for each value of Δ between 5 and 20 km. Both figures use the $M_{C}\geq$ 1.3.

Figure 14a shows the behavior of γ vs. different cubic cell sizes. It is possible to observe the same behavior as for the previous results: the value of γ decreases with growing cubic cells.

Table 1 shows an increase in the value of γ for all the values of Δ in the case in which the completeness magnitude was considered, with respect to the previous results. Values of γ are close to 2.0 for all the previous analyses and between 2.2 to 2.4 when $M_{c}$ is added. In this case, the error bars are larger for small values of Δ, between 5 to 8 km. However, the general behavior of the complex network remains: a structure with hubs for small values of Δ (few nodes with large degree) and a more homogeneous degree distribution for larger values of Δ.

#### 5.2.1. Southwestern Norway

**Figure 15:**
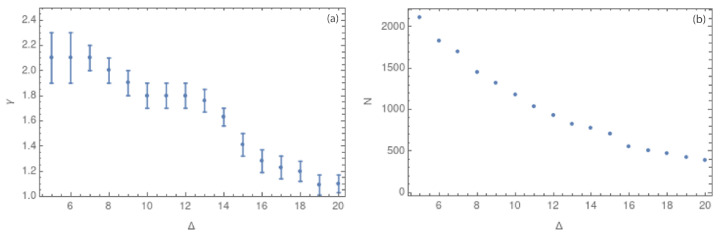
(**a**) Southwestern Norway: Values of the critical exponent γ in 3 dimensions for Δ between 5 and 20 km (**b**) Values of the number of nodes *N* for each value of Δ between 5 and 20 km. Both figures use the $M_{c}$.

The intraplate seismicity in Norway is varied in terms of causes (Figure 1). The seismicity patterns, if any, are still unknown, and the seismic events seem to behave in different ways according to the region, with large influence of regional variation in geological structures and stress fields [4,17]. For example, it has been established that seismicity in the southwestern coast is related to a high degree of weakness in the area, which strongly depends on the high degree of observed fracturing [17]. To determine if these behaviors are reflected in the complex network parameters, we analyzed 3111 seismic events located in the western zone (longitude 3.0° to 7.5° E). The results are shown in Figure 15.

#### 5.2.2. Southeastern Norway

Seismicity in southeastern Norway is predominantly linked to the Oslo region. This and adjacent areas were exposed to stretching and rifting between 359 and 252 Ma ago (Late Carboniferous–Early Permian). The rifting process implied high levels of magmatism, volcanism, and seismic activity. Traces of this intense activity include, for example, the main bodies of igneous rocks, which can be found inside the Oslo Graben [59]. The rifting process, which stopped 65 Ma ago (Cretaceous), left behind several tectonic episodes. Some of these are related to the emplacement of large intrusive bodies, which created a set of extensional structures like normal faults and grabens [59]. Nowadays, the seismic activity in the area can be partially linked to these faults [16].

**Figure 16:**
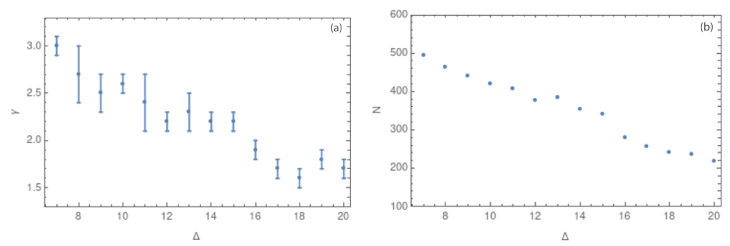
(**a**) Southeastern Norway: Values of the critical exponent γ in 3 dimensions for Δ between 5 and 20 km (**b**) Values of the number of nodes *N* for each value of Δ between 5 and 20 km. Both figures use the $M_{c}$.

The Oslo region subset has 628 seismic events after filtering (longitude 7.5° to 12.0° E). In this case, the scale-free behavior of the degree distribution can be analyzed only for side sizes starting from 7 km due to the small number of degrees for the 5 and 6 km cases. Figure 16a show the values of γ.

If we compare both areas, it is possible to observe differences between these two complex networks (Table 2). In the case of southwestern Norway, the behavior is the same as that we have found before: a structure of hubs. However, for the case of southeastern Norway, we found larger values of γ, and the slope did not decrease as fast as in the case of the western region. In this sense, the southeastern region clearly shows a different complex network structure.

Table 2 shows the γ values for the southwestern and the southeastern sub-catalogs in the first and second columns, respectively. It is easy to note that the values of the southwestern zone are smaller than the values for the southeastern zone. However, this might be because the quantity of data used to make the complex network analysis was smaller for the southeastern zone. To determine if the larger values of the critical exponent are influenced by the quantity of data, we repeated the same analysis for a small temporally sorted subset of data for the southwestern region. The results are shown in the third column of Table 2. The value of γ for the 628 seismic events of the southwestern zone is very similar to the results for the total of 3111 seismic events in the same zone, but with larger error bars. This result suggests that the larger values of γ obtained in the southeastern zone are due to the seismic environment of this zone and not due to the small quantity of data considered for this analysis. In fact, this result shows that the southeastern region has a smaller range of degrees than the southeastern region, which could suggest the presence of a larger number of hubs in the Oslo area.

## 6. Discussion and Conclusions

We used the method developed by Abe and Suzuki (2006) [39] to analyze the scale used to build nodes in a spatial earthquake complex network. To do so, we placed emphasis on the degree distribution of the nodes in the network, which is a well-known and extensively used measure in complex analyses. For earthquakes, it has been previously shown that the behavior of the degree distribution is a power law in a log-log plot of P(k) versus *k*, where *k* is the degree [39,43,45]. The slope of this power law is the critical exponent γ. In this study, our focal point is the analysis of γ, examining the reliability of the cell size that is used to define the nodes in this spatial complex network.

The analysis was performed using the hypocenters of seismic events for prefiltered and complete datasets, which, in this context, means without and with the magnitude of completeness, $M_{c}=1.3$. Figure 8a show how the value of the critical exponent depends on the side size of the cubic cell, which is called Δ. In this figure, error bars decrease with increasing Δ, a fact that could suggest a better adjustment for larger values of Δ. Figure 8b shows a decreasing number of nodes for larger Δ values.

In order to analyze the influence of the observational errors, Figure 9 shows the average error for each spatial coordinate: longitude, latitude, and depth. In groups (a) and (b) in Figure 9, the errors in longitude and latitude are negligible with respect to the change in the hypocentral longitude and latitude values in the studied area. However, the average error in depth is larger and considerable when compared to the average depth hypocentral values. To explore the influence of the error in depth on the obtained results, we also consider the complex network in two dimensions, i.e., (latitude, longitude). Figure 10a,b show the values of γ and the number of nodes for values of Δ between 5 and 20 km, respectively. Here, we can observe the same behavior that we found for the 3-dimensional case: the error bars decrease as Δ grows. However, for the largest values of Δ, the network structure is lost due to the homogenization of the degree distribution. Then, the best range of Δ is defined to be between 8 and 16 km.

Figure 11 shows the values of the critical exponent γ for 2D and 3D. It is interesting to note how the γ values do not seem to be affected by the change in dimension. This result is not conclusive, so we performed new analyses using a temporal subset of the data to verify if the quantity of data could affect the γ value. Figure 12a shows the behavior of γ for half of the seismic events, showing very similar results to those obtained before. Table 1 summarizes these three results, showing how the values of γ are very similar between them for all the cases, presenting great stability under a change of dimension or the number of events considered. The values obtained for Δ=5,10 and 20 km are close to 2, 1.4, and 1.2, respectively. These results suggest that the topology of the complex network changes with the values of Δ: the complex network built with Δ=5 km shows a structure with central nodes; meanwhile, with Δ=20 km, the values of γ are associated with a more homogeneous distribution of the degree in the complex network. Although these first results are not conclusive concerning the influence of measurement errors, they clearly show that nodes with smaller delta sizes can provide information about the complex network topology.

Since the previous results failed to provide clear information about the physics of intraplate seismic events, we performed a second analysis for the same dataset, but using the magnitude of completeness. Figure 14a and Table 1 summarize these results. In Table 1, it is possible to observe how the value of γ increases when the magnitude of completeness is used. For Δ between 5 and 7 km, the value of γ is greater than 2.0, and Δ between 9 and 15 km shows values of γ close to 2.0. For Δ between 16 and 20 km, the values of γ decrease and fluctuate between 1.35 and 1.54, showing again a more homogeneous distribution of the node degree. These results show the different behaviors of the complex network when the magnitude of completeness is used: the filtered low-magnitude events improve the complex network connection. When removed, the network has fewer nodes with a higher degree.

We have additionally compared the values obtained for Norway with the values computed for other regions of the planet, with different cubic cell sizes and using the same method. Table 3 summarizes these values. It is possible to observe that the values obtained by Abe and Suzuki [39] are smaller than the values obtained by Pastén et al. [40]. This difference may be because some results published by Abe and Suzuki do not consider the magnitude of completeness. However, the values of γ seem to follow the same behavior: larger γ values for smaller values of Δ. The values for Japan and Chile show similar behavior. In the Chilean case, the results before the occurrence of a large earthquake are more similar to the results obtained for the case of Norway, but with values 5 km lower than those found for Norwegian seismicity. California and Iran show the greatest difference from the other seismic regions. Although our intention in presenting these results was to initiate discussions in terms of the comparison between the different γ values, we finally conclude that there is no universality in the results. This means that some complex network parameters, for example, the one analyzed in this study, are strongly dependent on the seismicity type.

Finally, in order to understand the relevance of these results for the physical processes of intraplate seismicity in southern Norway, we added an extra analysis considering the spatial distribution of earthquakes in terms of longitude. This criterion is not random as the geological structures, the seismicity rates, the stress field, and the fracturing levels vary between the western and the eastern south Norwegian coasts, as explained before. The values of γ are greater for the Oslo region than in the zone of southwestern Norway, showing behavior where there are few nodes with a larger degree and more nodes with a small degree, as a tree structure with main hubs. This corresponds to an advance in linking the behavior of these parameters of complex networks with the physics of intraplate seismicity.

**Table 3:** Different results for the γ value obtained for different authors at several tectonic settings.

Place	Δ (km)	γ	Ref.
California	5	1.61	Abe and Suzuki 2006 [39]
	10	1.33	Abe and Suzuki 2006 [39]
	20	1.28	Abe et al. 2011 [43]
Japan	5	2.5	Abe and Suzuki 2006 [39]
	10	2.22	Abe and Suzuki 2006 [39]
	20	1.40	Abe et al. 2011 [43]
Iran	20	2.01	Abe et al. 2011 [43]
Chile	20	1.35	Abe et al. 2011 [43]
Chile (Illapel earthquake)	5	3.0 (before earthquake)	Pastén et al. 2016 [40]
Chile (Illapel earthquake)	10	2.2 (before earthquake)	Pastén et al. 2016 [40]
Chile (Illapel earthquake)	5	3.6 (after earthquake)	Pastén et al. 2016 [40]
Chile (Illapel earthquake)	10	2.2 (after earthquake)	Pastén et al. 2016 [40]

This is the first complex network-related study that has been carried out with seismic data recorded in Norway, so this research constitutes the first step to associate measurements of complex networks with the underlying physics involved in the occurrence of earthquakes, especially in this area of the planet and for this intraplate seismicity environment. All the constructed networks show stable behavior, which is replicated for the prefiltered catalog, the complete catalog, and the subsets. Even though we could characterize the complex network with all the above-mentioned variations, we can conclude that it is not easy to determine the best range of delta values. As a tentative conclusion, it seems better to analyze the complex network using 5, 10, and 15 km cell sizes, as larger values homogenize the network. Now, we will expand this study by calculating the clustering coefficient, different measures of centrality, and their associated critical exponents. 

## Figures and Tables

**Table 1:** Values of the critical exponent γ in 3D, 2D, for half of the seismic events and for the $M_{c}$, respectively.

Δ	γ 3D	γ 2D	γ Half Data	$M_{C}$
5 km	2.1 ± 0.1	2.2 ± 0.1	2.1 ± 0.1	2.4 ± 0.2
6 km	2.1 ± 0.1	2.0 ± 0.1	2.1 ± 0.1	2.3 ± 0.2
7 km	2.1 ± 0.1	1.83 ± 0.09	2.0 ± 0.1	2.2 ± 0.2
8 km	1.9 ± 0.1	1.74 ± 0.09	1.9 ± 0.1	2.1 ± 0.2
9 km	1.7 ± 0.1	1.73 ± 0.09	2.1 ± 0.1	2.0 ± 0.1
10 km	1.7 ± 0.1	1.6 ± 0.1	1.9 ± 0.1	2.0 ± 0.1
11 km	1.7 ± 0.09	1.6 ± 0.1	1.9 ± 0.1	1.9 ± 0.1
12 km	1.6 ± 0.1	1.52 ± 0.07	1.7 ± 0.1	2.0 ± 0.1
13 km	1.6 ± 0.1	1.47 ± 0.07	1.7 ± 0.1	1.8 ± 0.1
14 km	1.56 ± 0.08	1.43 ± 0.08	1.6 ± 0.1	1.8 ± 0.1
15 km	1.45 ± 0.08	1.38 ± 0.06	1.54 ± 0.08	1.8 ± 0.1
16 km	1.46 ± 0.06	1.38 ± 0.06	1.55 ± 0.08	1.54 ± 0.08
17 km	1.36 ± 0.07	1.35 ± 0.07	1.30 ± 0.09	1.45 ± 0.08
18 km	1.37 ± 0.06	1.27 ± 0.07	1.34 ± 0.07	1.46 ± 0.08
19 km	1.29 ± 0.06	1.26 ± 0.06	1.32 ± 0.08	1.41 ± 0.08
20 km	1.24 ± 0.05	1.17 ± 0.06	1.24 ± 0.08	1.35 ± 0.08

**Table 2:** Values of the critical exponent γ for southwestern Norway, southeastern Norway and for 628 seismic events in southwestern Norway.

Δ	γ SW Norway	γ SE Norway (Oslo)	γ Subset SW Norway
5 km	2.1 ± 0.1	–	—
6 km	2.1 ± 0.1	–	—
7 km	2.1 ± 0.1	3.0 ± 0.1	1.9 ± 0.4
8 km	2.0 ± 0.1	2.7 ± 0.2	1.8 ± 0.4
9 km	1.9 ± 0.1	2.5 ± 0.2	1.9 ± 0.3
10 km	1.7 ± 0.1	2.6 ± 0.2	1.9 ± 0.3
11 km	1.8 ± 0.1	2.4 ± 0.2	1.7 ± 0.3
12 km	1.8 ± 0.1	2.2 ± 0.2	1.7 ± 0.3
13 km	1.76 ± 0.09	2.3 ± 0.2	1.7 ± 0.2
14 km	1.63 ± 0.08	2.2 ± 0.1	1.7 ± 0.2
15 km	1.48 ± 0.09	2.2 ± 0.1	1.7 ± 0.2
16 km	1.28 ± 0.09	1.9 ± 0.1	1.5 ± 0.2
17 km	1.23 ± 0.09	1.7 ± 0.1	1.5 ± 0.1
18 km	1.20 ± 0.08	1.7 ± 0.1	1.4 ± 0.2
19 km	1.09 ± 0.08	1.8 ± 0.1	1.4 ± 0.1
20 km	1.10 ± 0.07	1.8 ± 0.1	1.4 ± 0.1

**Figure A1:**
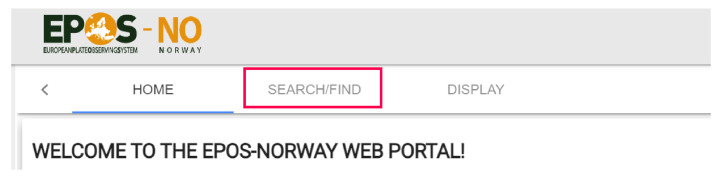
Capture from https://epos$-$no.uib.no/eposn$-$data$-$portal/ (accesed on 12 January 2023).

**Figure A2:**
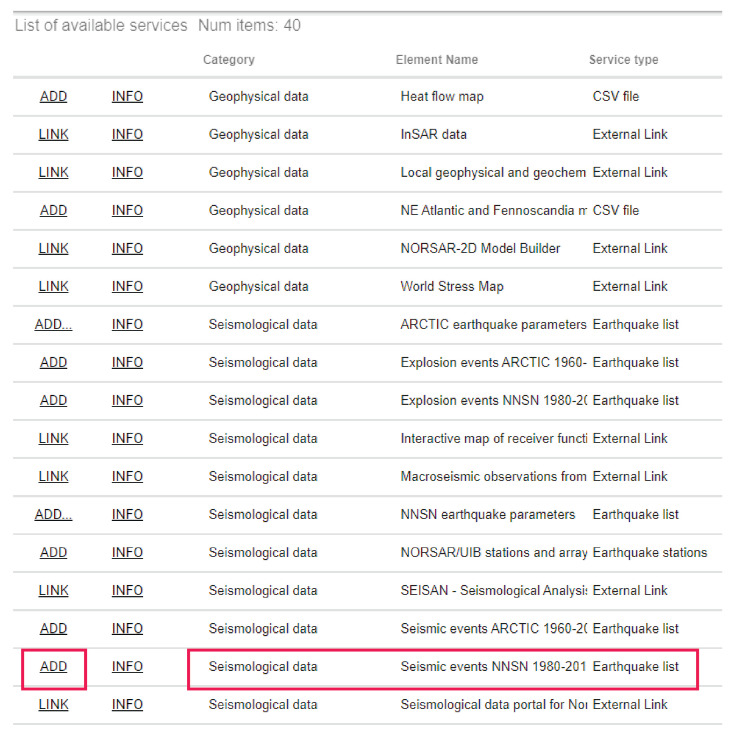
Capture from https://epos$-$no.uib.no/eposn$-$data$-$portal/ (accesed on 12 January 2023).

**Figure A3:**
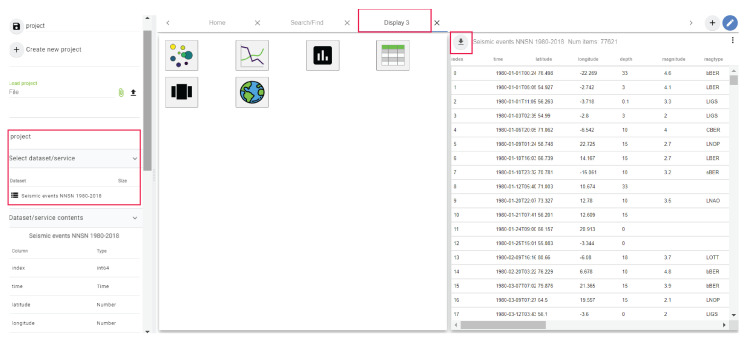
Capture from https://epos$-$no.uib.no/eposn$-$data$-$portal/ (accesed on 12 January 2023).

## Data Availability

This publication used open and public data, available at URL http://www.epos-no.org/ (accesed on 12 January 2023). For more details please follow the instructions detailed on Appendix A.

## Appendix A

The instructions to download the original catalogue as further processed to be used in this study are presented here.

After entering the EPOS-N portal, the SEARCH/FIND option must be selected (Figure A1).

Then, a list of around 40 different data services will appear on the right-hand side of the screen. The option to download the NNSN seismological catalog is located at the end of the list (Figure A2). By pressing the ADD button (Figure A2), the data will be added to the project (Figure A3).

Using the DISPLAY option, the data can be visualized in several formats. As shown in (Figure A3), the options include map, linear, and histogram plotting, among others. The catalog will also be available in .csv format to be downloaded and processed (Figure A3).
